# Conventional methods to prescribe exercise intensity are ineffective for exhaustive interval training

**DOI:** 10.1007/s00421-023-05176-6

**Published:** 2023-03-29

**Authors:** Arthur Henrique Bossi, Diana Cole, Louis Passfield, James Hopker

**Affiliations:** 1 School of Sport and Exercise Sciences, University of Kent, Canterbury, Kent, UK; 2 School of Applied Sciences, Edinburgh Napier University, Edinburgh, UK; 3 The Mountain Bike Centre of Scotland, Peel Tower, Glentress, Peebles, EH45 8NB UK; 4 School of Mathematics, Statistics and Actuarial Science, University of Kent, Canterbury, Kent, UK; 5 Faculty of Kinesiology, University of Calgary, Calgary, AB Canada

**Keywords:** Intermittent exercise, Individual response, Delta concept, Adaptive variability, Trainability

## Abstract

**Purpose:**

To compare methods of relative intensity prescription for their ability to normalise performance (i.e., time to exhaustion), physiological, and perceptual responses to high-intensity interval training (HIIT) between individuals.

**Methods:**

Sixteen male and two female cyclists (age: 38 ± 11 years, height: 177 ± 7 cm, body mass: 71.6 ± 7.9 kg, maximal oxygen uptake ($\dot{\text{V}}$O_2max_): 54.3 ± 8.9 ml·kg^−1^ min^−1^) initially undertook an incremental test to exhaustion, a 3 min all-out test, and a 20 min time-trial to determine prescription benchmarks. Then, four HIIT sessions (4 min on, 2 min off) were each performed to exhaustion at: the work rate associated with the gas exchange threshold ($\dot{\text{W}}$_GET_) plus 70% of the difference between $\dot{\text{W}}$_GET_ and the work rate associated with $\dot{\text{V}}$O_2max_; 85% of the maximal work rate of the incremental test (85%$\dot{\text{W}}$_max_); 120% of the mean work rate of the 20 min time-trial (120%TT); and the work rate predicted to expend, in 4 min, 80% of the work capacity above critical power. Acute HIIT responses were modelled with participant as a random effect to provide estimates of inter-individual variability.

**Results:**

For all dependent variables, the magnitude of inter-individual variability was high, and confidence intervals overlapped substantially, indicating that the relative intensity normalisation methods were similarly poor. Inter-individual coefficients of variation for time to exhaustion varied from 44.2% (85%$\dot{\text{W}}$_max_) to 59.1% (120%TT), making it difficult to predict acute HIIT responses for an individual.

**Conclusion:**

The present study suggests that the methods of intensity prescription investigated do not normalise acute responses to HIIT between individuals.

## Introduction

The prescription of endurance training involves decisions about intensity, duration, frequency, and mode of exercise. Of these variables, exercise intensity is arguably the most challenging to prescribe. This difficulty stems from the fact that a given work rate may elicit various levels of cardiorespiratory and metabolic stress depending on the individual’s physiological capacity. Therefore, the first step in prescribing exercise training is to decide on a test that provides a benchmark to be used for the normalisation of relative intensity.

An incremental test to exhaustion has typically been the preferred method to measure the maximal oxygen uptake ($\dot{\text{V}}$O_2max_) as an index of cardiorespiratory fitness (Hawkins et al. 2007). Likewise, using fractions of an individual’s $\dot{\text{V}}$O_2max_ to express exercise intensity (%$\dot{\text{V}}$O_2max_) has been a common practice for decades (Åstrand and Ryhming 1954). However, there are criticisms of this approach. Some studies suggest that using %$\dot{\text{V}}$O_2max_ to prescribe exercise may elicit highly heterogeneous responses between individuals (e.g., blood lactate concentration ([La^−^]), ratings of perceived exertion (RPE), time to exhaustion) (Scharhag-Rosenberger et al. 2010; Coyle et al. 1988; Lansley et al. 2011; McLellan and Skinner 1985; Meyer et al. 1999; Baldwin et al. 2000; Egger et al. 2016; Iannetta et al. 2020). Therefore, the effectiveness of training programmes based on %$\dot{\text{V}}$O_2max_ would likely be compromised in those individuals experiencing less homeostatic perturbations, given the role of relative exercise intensity in activating signalling pathways that mediate physiological adaptation (MacInnis and Gibala 2017; Granata et al. 2018). Such a prospect indicates that alternative benchmarks should be considered for exercise intensity normalisation.

Mann et al. (2013) have reviewed some of the methods to prescribe exercise intensity described in the literature. No consensus emerged as to the best method (Mann et al. 2013), with a similar conclusion being reached by a more recent review (Jamnick et al. 2020). It has been argued that the optimal method of intensity prescription may be context dependent, as the population of interest, targeted intensity domain (i.e., moderate, heavy, very heavy, or severe; see Rossiter (2011)), and exercise pattern (i.e., continuous or intermittent) are likely to determine the ideal choice (Mann et al. 2013; Jamnick et al. 2020). For this reason, it seems counterintuitive that few studies have investigated exercise intensity normalisation for high-intensity interval training (HIIT) (Julio et al. 2020; Galbraith et al. 2015; Ferguson et al. 2013; Bartram et al. 2018). In this type of training, exercise is performed intermittently at work rates that can be sustained for only a few minutes, due to the energetic demand exceeding the capacity of muscle cells to synthesise adenosine triphosphate (Rossiter 2011). Performance variability at such intensities can be explained by the critical power (CP) model (Jones and Vanhatalo 2017), and it is therefore unsurprising that most HIIT studies on exercise intensity normalisation have investigated this framework (Ferguson et al. 2013; Galbraith et al. 2015; Bartram et al. 2018). However, in a previous study with runners (Galbraith et al. 2015), and a further investigation with elite cyclists (Bartram et al. 2018), HIIT performance predictions based on the CP model proved inaccurate, posing a challenge to practitioners and researchers.

While it may be difficult to normalise exercise intensity for HIIT across individuals, it has been generally assumed that longitudinal HIIT interventions elicit adaptive variability between participants (Astorino and Schubert 2014; Coakley and Passfield 2018; Montero and Lundby 2017; Williams et al. 2019). It could be argued that this outcome results, at least in part, from the methodology associated with how training work rates are set for each participant (Mann et al. 2014, 2013; Iannetta et al. 2020; Jamnick et al. 2020). Therefore, the purpose of this study was to compare methods of intensity prescription for their ability to normalise performance (i.e., time to exhaustion), physiological, and perceptual responses to HIIT between individuals. Four existing methods were chosen according with their standing in the scientific literature, which are based on the following benchmarks: the delta between gas exchange threshold and $\dot{\text{V}}$O_2max_ (Lansley et al. 2011), the maximal work rate of an incremental test to exhaustion ($\dot{\text{W}}$_max_) (Granata et al. 2018), the mean work rate of a 20 min time-trial (Nimmerichter et al. 2011), and the work capacity above CP (W′) (Jones and Vanhatalo 2017). It was hypothesised that there would a between-method difference in the magnitude of inter-individual variability in acute HIIT responses.

## Methods

### Participants

Sixteen male and two female recreationally trained competitive cyclists (age: 38 ± 11 years, height: 177 ± 7 cm, body mass: 71.6 ± 7.9 kg, cycling experience index: 26 ± 5 (see Edwards et al. (2009) for details) volunteered for this study. The research protocols were submitted to, and approved by the Research Ethics Committee at the University of Kent, in compliance with the Declaration of Helsinki. Prior to participation, written informed consent was obtained from all participants.

### Study design

**Fig. 1:**
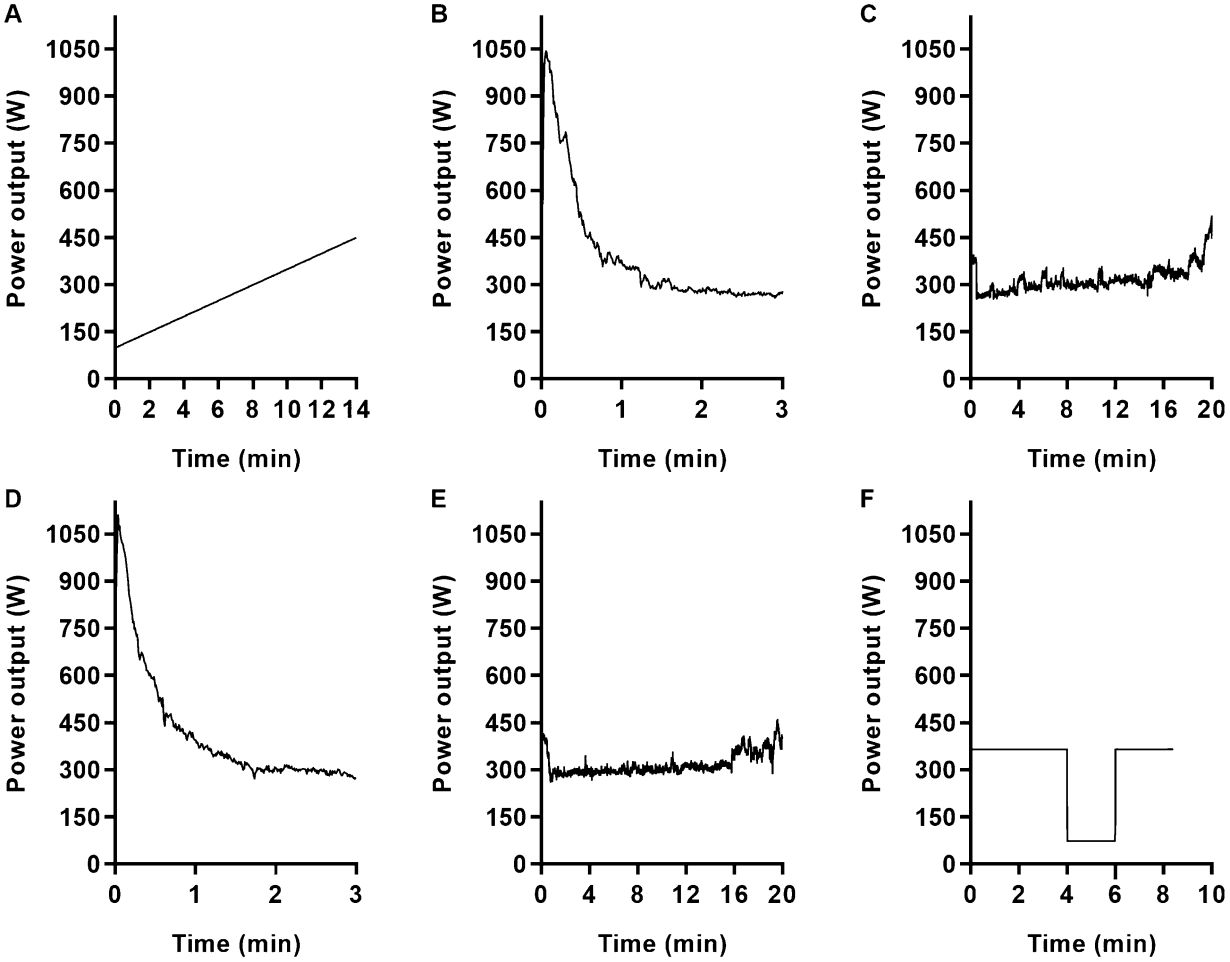
Power output of a representative participant to illustrate the tests performed in the first (panel A: incremental test, panel B: 3 min all-out test, panel C: 20 min time-trial) and second visits (panel D: 3 min all-out test, panel E: 20 min time-trial, panel F: high-intensity interval training session). In the first visit, the 3 min all-out test and the 20 min time-trial were performed as a familiarisation. In the second visit, the high-intensity interval training session was performed as a familiarisation

Participants attended the laboratory on six occasions, at the same time of the day, separated by at least 48 h. In the first visit (Fig. 1, panels A, B, and C), they completed an incremental test to exhaustion, a 3-min all-out test, and a 20 min time-trial, sequentially. The last two tests were performed as a familiarisation. In the second visit (Fig. 1, panels D, E, and F), the 3 min all-out test and the 20 min time-trial were initially repeated in this order for data collection purposes. Next, a HIIT session was performed to exhaustion, to familiarise participants with the training format of subsequent visits. Thereafter, participants performed a HIIT session to exhaustion per visit, with different intensity normalisation methods randomly allocated to each of the four visits (see text below for details). Inter-individual variability in performance, physiological and perceptual responses were compared between HIIT sessions.

All tests started with a 10 min warm-up at 100 W for men, and 50 W for women, except for HIIT sessions (see text below for details). In the first and second visits, tests were separated by 10 min of low-intensity cycling followed by 20 min of rest. Participants were instructed to refrain from all types of intense exercise 48 h before laboratory visits and to prepare as they would for competition. They were requested to standardise meals 24 h before each visit, and to record them on a food diary to enhance compliance. The consumption of caffeine was not allowed in the last 24 h before testing. All tests were performed free from distractions, under similar environmental conditions (16–17 °C), with participants being cooled with a fan. Strong encouragement was provided to warrant representative performances.

### Equipment

Cyclists used their own bikes mounted on a cycle ergometer (Cyclus 2, RBM Elektronik-Automation, Leipzig, Germany). For the incremental test and HIIT sessions, the ergometer was set at power mode (i.e., cadence independent). For the 3 min all-out tests and 20 min time-trials, the ergometer was set at inclination mode (i.e., 0%; cadence dependent), and participants were required to change the gears of the bike, as if they were riding outdoors. Elapsed time and cadence were always visible, except for the 3 min all-out tests. In contrast, power output was visible only during the 20 min time-trials. Heart rate was continuously monitored during all sessions through an ANT + belt transmitter (Cyclus 2, RBM Elektronik-Automation, Leipzig, Germany), but data were concealed from participants.

Breath-by-breath gas exchange was continuously monitored through a metabolic cart (MetaLyzer 3B, Cortex Biophysik, Leipzig, Germany) during the incremental test and HIIT sessions. Prior to every test, calibration was performed according with the manufacturer’s instructions. Fingertip capillary blood samples were assessed for [La^−^] in an automatic analyser (Biosen C-Line, EKF Diagnostics, Penarth, UK). RPE was assessed based on the 6–20 Borg’s scale (Borg 1982).

Changes from a 3-min resting baseline in muscle tissue oxygen saturation (ΔStO_2_) and concentration of deoxygenated heme compounds (Δdeoxy[heme]) were assessed during HIIT sessions using continuous-wave near-infrared spectroscopy (Portamon, Artinis Medical Systems, Elst, Netherlands) at a sampling rate of 10 Hz. The near-infrared spectroscopy signal for deoxygenated haemoglobin and myoglobin was multiplied by four to express data as units of heme (Barstow 2019). The inter-optode distance was 35 mm and a differential path-length factor of 4.0 was assumed for all tests. The device was placed on the vastus lateralis above the upper patella border, at one-third of the distance between the patella and the greater trochanter, parallel to the longitudinal femur axis. This site was shaved, and adipose tissue thickness was estimated by skinfold callipers as the halved median of three measurements (3.6 ± 2.3 mm) (Barstow 2019). Motion artefacts were minimised by fixating the device position using a cohesive compression bandage. A plastic wrap and a light-absorbing black cloth were used to cover the apparatus. Position was marked for replication in subsequent measurements.

### Incremental test

Immediately after the warm-up, work rate increased continuously at 25 W min^−1^ until voluntary exhaustion, or participants’ inability to maintain cadence above 70 rev min^−1^. $\dot{\text{V}}$O_2max_ was identified as the highest 30 s mean oxygen uptake, and $\dot{\text{W}}$_max_ as the mean power output of the last minute. Gas exchange threshold (GET) was obtained according with the procedures described by Lansley et al. (2011), as the first disproportionate increase in carbon dioxide output vs. oxygen uptake; an increase in ventilatory equivalent for oxygen with no increase in ventilatory equivalent for carbon dioxide; and an increase in end-tidal oxygen tension with no fall in end-tidal carbon dioxide tension. Two-thirds of the ramp rate were deducted from the work rate at GET to account for the oxygen uptake response time. Immediately after the incremental test, a blood sample was taken from a fingertip to establish [La^−^], and peak RPE was noted.

### 3-min all-out test

In the first visit only, following the warm-up, participants were given the chance to practice two 5-s all-out sprints to select the best gear to start the test. A 5 min active recovery was allowed after sprints. Immediately before the test, participants were required to pedal slowly at the optimal gear for 5 s (~ 150 W). On command, they started an all-out effort for 3 min, with gears being minimally changed (2 or 3 times), always towards the next bigger cog. Participants were not informed of elapsed time to prevent pacing, but strong verbal encouragement was provided throughout the test, which was terminated at 185 s to ensure that a full 3 min effort was completed. CP was estimated from the mean power output between 150 and 180 s, and W' from the power output–time integral above CP (Vanhatalo et al. 2007).

### 20 min time-trial

Immediately after the warm-up, participants started the time-trial with the aim of producing the highest possible mean power output for 20 min. They were instructed on how to optimise pacing by observing the graphical feedback on the ergometer screen. Participants drank water and stood on the pedals as desired.

### Intensity prescription

**Fig. 2:**
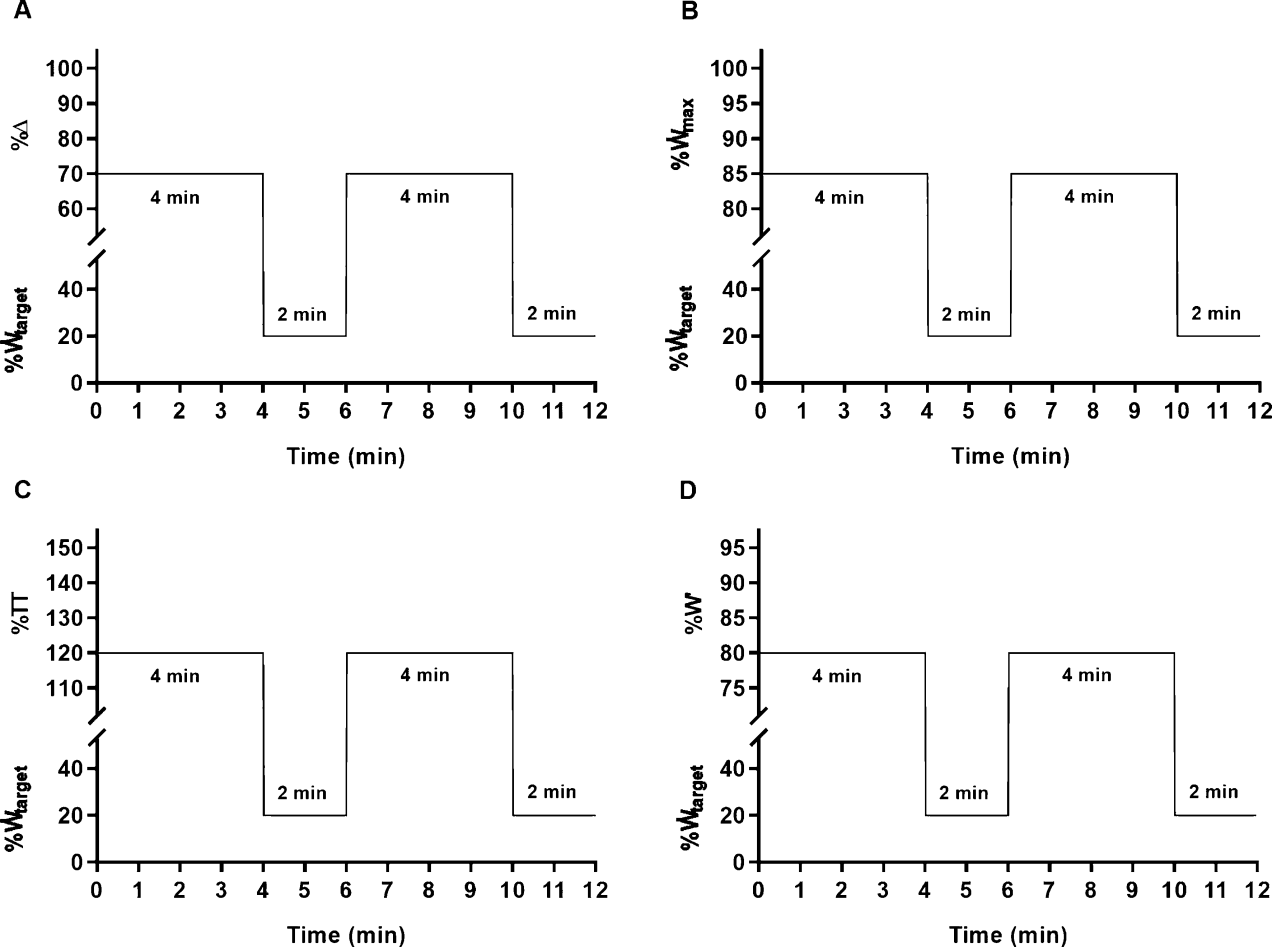
Schematic representation of the high-intensity interval training sessions that participants performed to exhaustion at 70%Δ (panel A), 85%$\dot{\text{W}}$_max_ (panel B), 120%TT (panel C), and 80%W' (panel D), randomly, on four separate occasions. See text for intensity prescription abbreviations. $\dot{\text{W}}$_target_ represents the work rate prescribed for each condition. An identical 10 min warm-up, followed by a two-minute resting period, preceded all sessions. For clarity, these are omitted and only two work intervals are represented

Four intensity normalisation methods were used to set the work rate for the work intervals of each HIIT session: a) the work rate associated with GET ($\dot{\text{W}}$_GET_) plus 70% of the difference between $\dot{\text{W}}$_GET_ and the work rate associated with $\dot{\text{V}}$O_2max_ (70%Δ – Fig. 2A); b) 85% $\dot{\text{W}}$_max_ (Fig. 2B); c) 120% of the mean work rate of the 20 min time-trial (120%TT – Fig. 2C); d) the work rate predicted to expend 80%W' in 4 min, according with:

1 $$ \dot{\text{W}}_\text{target} = 0.8{\text{W}}^{' } /240 + {\text{CP}} $$

where $\dot{\text{W}}$_target_ is the work rate prescribed, W' is the work capacity above critical power, and CP is critical power (Fig. 2D). Recovery intervals were always performed at 20% of the work rate prescribed for the work intervals.

To ensure that HIIT intensity was comparable on average, the percentage of each prescription benchmark was derived based on pilot work with an independent sample of five male cyclists (age: 28 ± 3 years, height: 173 ± 10 cm, body mass: 66.3 ± 11.2 kg, $\dot{\text{V}}$O_2max_: 59.2 ± 7.1 ml·kg^−1^·min^−1^). The work rates for 70%Δ, 85%$\dot{\text{W}}$_max_, 120%TT, and 80%W' corresponded to 4.59 ± 0.76, 4.57 ± 0.66, 4.61 ± 0.65, and 4.67 ± 0.80 W·kg^−1^, respectively (*F* = 0.41, *P* = 0.62, *ƞ*_*p*_^2^ = 0.09).

### HIIT sessions

The same 10 min warm-up was performed before every HIIT session. Two 5 min bouts were performed sequentially at 40 and 50% of the mean work rate prescribed for the work intervals of all four HIIT sessions. The first work interval started 2 min after the warm-up was terminated. During this resting period, the metabolic cart was set up and participants wore the facemask. In the last 10 s before HIIT sessions started, participants increased cadence to > 100 rev·min^−1^. Work intervals of 4 min, interspersed with active recoveries of 2 min, were repeated to exhaustion or until ten work intervals were completed (participants were not aware of this arbitrary endpoint). Cadence was self-selected during both work and recovery intervals and exhaustion was defined with the same criteria as the incremental test. RPE was indicated immediately after each work interval and at exhaustion. Blood samples for the assessment of [La^−^] were collected 20 s into the recovery intervals, and 20 s after exhaustion. Ten minutes after the HIIT sessions, session RPE (sRPE) was recorded (Foster et al. 2001).

### Questionnaires

Upon arrival at the laboratory, participants answered a series of questions to determine their cycling experience (Edwards et al. 2009) (first visit only), intrinsic and success motivations (Matthews et al. 2001), and sport emotions (i.e., anxiety, dejection, excitement, anger, and happiness) (Jones et al. 2005). They also indicated their sleep duration, and rated from 1 to 10 their sleep quality, motivation to train, appetite, overall recovery status, muscle soreness, how heavy they were feeling, and how heavy their legs were feeling. These latter scales were adapted from a previous version of the Norwegian Olympic Committee’s training diary (http://olt-dagbok.nif.no) and are hereafter referred to as training diary scales. At the end of each exercise session, participants rated subjective workload using the National Aeronautics and Space Administration Task Load Index (NASA-TLX) composed of six subscales: mental demand, physical demand, temporal demand, performance, effort, and frustration (Hart 2006). In the morning after each laboratory visit, participants indicated their sleep duration and completed the training diary scales once more. Questionnaires and scales were administered in the first and second visits for familiarization purposes only.

### Data processing

Raw breath-by-breath gas data were smoothed to 5-s averages. Time > 90% $\dot{\text{V}}$O_2max_ and time > 95% $\dot{\text{V}}$O_2max_ were calculated for each HIIT session by summing all oxygen uptake samples above the established cut-off. Time > 90% $\dot{\text{V}}$O_2max_ and time > 95% $\dot{\text{V}}$O_2max_ were also calculated as a percentage of time to exhaustion (i.e., time > 90% $\dot{\text{V}}$O_2max[%TTE]_ and time > 95% $\dot{\text{V}}$O_2max[%TTE]_, respectively). Cadence was analysed as the average of each work interval. Oxygen uptake, heart rate, ventilation, respiratory frequency, ΔStO_2_, and Δdeoxy[heme] were analysed as the average of the last minute of each work interval, or the completed duration if shorter than one minute, although maximal responses were sometimes elicited during the last complete work interval, but not during the incomplete one.

### Data analysis

Data were assessed for normality using the Shapiro–Wilk’s test and normal quantile plots. For the dependent variables conforming to a normal distribution, one-way repeated measures analyses of variance were performed to test for systematic differences between conditions (70%Δ, 85% $\dot{\text{W}}$_max_, 120%TT, or 80%W'), with Bonferroni pairwise comparisons used to identify where significant differences existed within the data. As time to exhaustion, time > 90% $\dot{\text{V}}$O_2max_, and time > 95% $\mathop {\text{V}}\limits^{.}$O_2max_ did not meet the normality assumption, Friedman analyses of variance were performed to investigate between-condition differences, with Dunn pairwise comparisons used to identify where significant differences existed within the data. To investigate the magnitude of inter-individual variability in time to exhaustion as a function of exercise intensity, all times were first log-transformed (base *e*). Then, the four target work rates calculated for each HIIT session (as 70%Δ, 85% $\dot{\text{W}}$_max_, 120%TT, and 80%W'), for each participant, were also expressed as %$\dot{\text{V}}$O_2max_, %Δ, % $\dot{\text{W}}$_max_, %TT, and %W'. Linear mixed models were fitted to the logarithm of time to exhaustion with relative intensity as a fixed factor and participant as a random effect. The inter-individual coefficient of variation (CV) for log-transformed time to exhaustion was calculated as:

2 $$ {\text{CV}}\left( \% \right) = \surd \left( {e^{Var - 1} } \right) $$

where Var is the variance of the log-transformed data. The relationship between both RPE and [La^−^] at the end of the first work interval and log-transformed time to exhaustion were assessed with correlational analysis adjusted for repeated observations within participants. Linear mixed models were also used to investigate the magnitude of inter-individual variability in oxygen uptake, RPE, [La^−^], heart rate, ventilation, respiratory frequency, ΔStO_2_, Δdeoxy[heme], and cadence, with participant as a random effect. When appropriate, work interval was considered as a fixed factor, which was either linear, quadratic, or cubic. No specific function was assumed, and the optimal model was selected using likelihood ratio tests. Ninety-five percent confidence limits were calculated by bootstrap sampling with 200 repetitions (Davison and Hinkley 1997). Systematic differences between conditions were assessed using Prism 8 (GraphPad, San Diego, USA) and data modelling was performed using R 4.0.4 (R Foundation for Statistical Computing, Vienna, Austria). Significance level was set at *P* ≤ 0.05. Results are presented as mean ± SD unless otherwise stated. When appropriate, partial eta squared is presented as a measure of effect size (*ƞ*_*p*_^2^). The reader unfamiliarised with linear mixed models is referred to Brown (2021) and Faraway (2016).

## Results

During the incremental test, participants attained a $\dot{\text{V}}$O_2max_ of 54.3 ± 8.9 ml·kg^−1^·min^−1^, $\dot{\text{W}}$_max_ of 5.01 ± 0.80 W·kg^−1^, $\dot{\text{W}}$_GET_ of 2.76 ± 0.46 W·kg^−1^, peak heart rate of 179 ± 14 beats·min^−1^, peak respiratory exchange ratio of 1.19 ± 0.05, [La^−^] of 12.0 ± 3.3 mmol·L^−1^, and RPE of 19.5 ± 0.5. Estimated CP and W' based on the 3-min all-out test were 3.72 ± 0.73 W·kg^−1^ and 215.7 ± 70.4 J·kg^−1^, respectively. The mean power output of the 20-min time-trial was 3.65 ± 0.60 W·kg^−1^. The work and recovery intervals of the HIIT sessions were performed, respectively, at 4.16 ± 0.65 and 0.83 ± 0.13 W·kg^−1^ for 70%Δ, 4.26 ± 0.68 and 0.85 ± 0.14 W·kg^−1^ for 85%$\dot{\text{W}}$_max_, 4.38 ± 0.72 and 0.88 ± 0.14 W·kg^−1^ for 120%TT, and 4.44 ± 0.82 and 0.89 ± 0.16 W·kg^−1^ for 80%W'. The warm-up bouts were performed at 1.72 ± 0.28 and 2.15 ± 0.34 W·kg^−1^.

No systematic differences between conditions were evident for time > 95% $\dot{\text{V}}$O_2max_ (*F*_*r*_ = 3.73, *P* = 0.29), time > 90% $\dot{\text{V}}$O_2max[%TTE]_ (*F* = 0.61, *P* = 0.56, *ƞ*_*p*_^2^ = 0.03), time > 95% $\dot{\text{V}}$O_2max[%TTE]_ (*F* = 0.32, *P* = 0.73, *ƞ*_*p*_^2^ = 0.02), sRPE (*F* = 2.48, *P* = 0.09, *ƞ*_*p*_^2^ = 0.13), intrinsic motivation (*F* = 0.05, *P* = 0.96, *ƞ*_*p*_^2^ = 0.00), success motivation (*F* = 1.60, *P* = 0.21, *ƞ*_*p*_^2^ = 0.09), sport emotions (all *F* ≤ 1.81, *P* ≥ 0.17, *ƞ*_*p*_^2^ ≤ 0.10), pre-HIIT sleep duration (*F* = 1.36, *P* = 0.27, *ƞ*_*p*_^2^ = 0.07), post-HIIT sleep duration (*F* = 0.27, *P* = 0.72, *ƞ*_*p*_^2^ = 0.02), training diary scales (all *F* ≤ 1.54, *P* ≥ 0.23, ƞ_p_^2^ ≤ 0.08), or the NASA-TLX subscales of mental demand (*F* = 1.44, *P* = 0.25, *ƞ*_*p*_^2^ = 0.08), physical demand (*F* = 0.80, *P* = 0.49, *ƞ*_*p*_^2^ = 0.04), temporal demand (*F* = 0.34, *P* = 0.70, *ƞ*_*p*_^2^ = 0.02), and effort (*F* = 0.63, *P* = 0.55, *ƞ*_*p*_^2^ = 0.04).

**Fig. 3:**
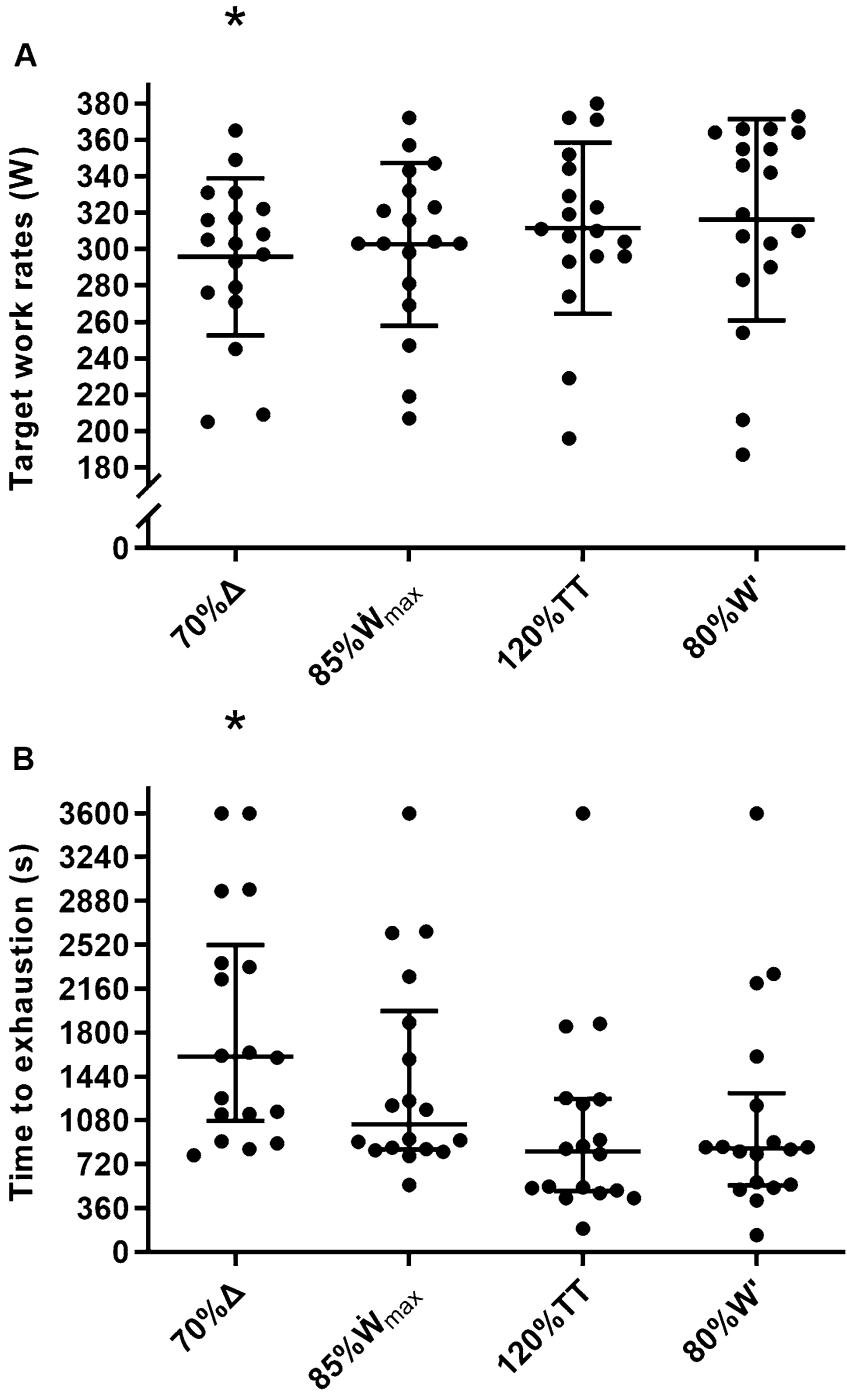
Target work rates for the work intervals of each high-intensity interval training session (panel A), and associated time to exhaustion (panel B). Horizontal bars represent the mean (panel A) and median (panel B), whiskers represent the standard deviation (panel A) and interquartile range (panel B), and dots represent individual values. Participants were stopped at 3600 s (i.e., end of the 10th work interval; see text for details). * denotes difference from all other conditions in panel A (all *P* ≤ 0.038), and difference from 120%TT and 80%W' in panel B (both *P* ≤ 0.022). See text for intensity prescription abbreviations

However, there was a condition effect for the power output (W·kg^−1^; for absolute power output, see Fig. 3A) at which work (*F* = 9.56, *P* < 0.001, *ƞ*_*p*_^2^ = 0.36) and recovery intervals (*F* = 9.09, *P* < 0.001, *ƞ*_*p*_^2^ = 0.35) were performed, time to exhaustion (*F*_*r*_ = 16.20, *P* = 0.001 – Fig. 3B), time > 90% $\dot{\text{V}}$O_2max_ (*F*_*r*_ = 10.00, *P* = 0.018), and the NASA-TLX subscales of performance (*F* = 3.86, *P* = 0.027, *ƞ*_*p*_^2^ = 0.19) and frustration (*F* = 6.46, *P* = 0.003, *ƞ*_*p*_^2^ = 0.28). Pairwise comparisons revealed that the power outputs (W·kg^−1^) at which work and recovery intervals were performed were lower for 70%Δ compared with all other conditions (all *P* ≤ 0.033). As a consequence, time to exhaustion was longer for 70%Δ compared with 120%TT and 80%W' (both *P* ≤ 0.022), while time > 90% $\dot{\text{V}}$O_2max_ was longer for 70%Δ compared with 120%TT only (*P* = 0.014). Performance was rated poorer for 120%TT compared with 70%Δ and 85%$\dot{\text{W}}$_max_ (both *P* ≤ 0.027), and frustration was rated higher for 80%W' compared with 70%Δ and 85%$\dot{\text{W}}$_max_ (both *P* ≤ 0.035).

The median times to exhaustion (25th percentile–75th percentile) were 26.7 min (17.9–42.0 min), 17.4 min (14.0–33.0 min), 13.8 min (8.3–20.9 min), and 14.2 min (9.1–21.7 min), for 70%Δ, 85%$\dot{\text{W}}$_max_, 120%TT, and 80%W', respectively. Estimates of inter-individual variability in log-transformed time to exhaustion as a function of exercise intensity are presented in Table 1. There were inverse correlations between both RPE (*r* = – 0.35, *r*^2^ = 0.12, *P* = 0.010) and [La^−^] (*r* = – 0.52, *r*^2^ = 0.27, *P* ≤ 0.001) at the end of the first work interval and log-transformed time to exhaustion. Estimates of inter-individual variability in physiological responses, RPE, and cadence for each HIIT condition are presented in Table 2. For all variables, confidence intervals overlapped substantially, indicating that all intensity normalisation methods elicited similar magnitudes of inter-individual variability. The magnitude of inter-individual variability in time > 90% $\dot{\text{V}}$O_2max[%TTE]_ and time > 95% $\dot{\text{V}}$O_2max[%TTE]_ was also similar between conditions (Table 3).

**Table 1:** Linear mixed model estimates for the natural logarithm of time to exhaustion [95% confidence limits]

Dependent variable	Intensity prescription	Intercept	Intensity coefficient	Inter-individual SD	Inter-individual CV (%)	Standard error of estimate	Inter-individual variability (%)	Residual variability (%)
log_*e*_ (time to exhaustion)	%$\dot{\text{V}}$O_2max_	20.1 [18.0–22.6]	– 0.142 [– 0.171 to – 0.120]	0.476 [0.290–0.649]	50.4 [29.6–72.4]	0.321 [0.258–0.369]	68.7 [44.7–82.5]	31.3 [17.0–54.8]
	%Δ	10.4 [9.8–11.0]	– 0.044 [– 0.052 to – 0.037]	0.503 [0.326–0.703]	53.7 [33.5–80.0]	0.335 [0.276–0.401]	69.2 [46.2–82.3]	30.8 [17.5–51.9]
	%$\dot{\text{W}}$_max_	17.0 [15.1–18.9]	– 0.116 [– 0.138 to – 0.096]	0.422 [0.271–0.576]	44.2 [27.6–62.7]	0.326 [0.259–0.389]	62.6 [37.1–77.1]	37.4 [22.8–59.4]
	%TT	16.6 [14.9–18.3]	– 0.082 [– 0.095 to – 0.067]	0.547 [0.318–0.740]	59.1 [32.6–85.4]	0.326 [0.267–0.385]	73.8 [46.1–85.1]	26.2 [14.8–51.8]
	%W'	8.1 [7.8–8.5]	– 0.017 [– 0.021 to – 0.013]	0.466 [0.265–0.688]	49.2 [27.0–77.8]	0.390 [0.309–0.461]	58.9 [31.4–75.7]	41.1 [22.9–65.7]

Formula: time to exhaustion (s) = 2.718282^(intercept + intensity coefficient · *x*%)^, where *x* = relative intensity. See text for intensity prescription abbreviations

**Table 2:** Linear mixed model estimates for acute responses to exhaustive interval training [95% confidence limits]

Dependent variable	Intensity prescription	Intercept	Interval coefficient_(a)_	Interval coefficient_(b)_	Interval coefficient_(c)_	Inter-individual SD	Standard error of estimate	Inter-individual variability (%)	Residual variability (%)
Oxygen uptake (ml·kg^−1^·min^−1^)	70%Δ	50.3 [47.4–53.2]	N/A	N/A	N/A	6.6 [4.1–9.3]	2.2 [1.9–2.6]	89.7 [76.2–94.8]	10.3 [5.2–23.4]
	85%$\dot{\text{W}}$_max_	50.5 [47.4–54.5]	N/A	N/A	N/A	7.4 [4.7–9.9]	2.4 [2.0–2.8]	90.7 [75.9–94.9]	9.3 [5.0–23.0]
	120%TT	50.1 [45.9–54.2]	N/A	N/A	N/A	8.3 [5.4–10.9]	2.5 [2.0–3.1]	91.4 [80.6–95.4]	8.6 [4.5–18.8]
	80%W'	51.2 [47.5–55.2]	N/A	N/A	N/A	8.2 [5.3–10.3]	2.4 [1.9–2.9]	92.3 [83.2–95.7]	7.7 [4.1–16.5]
Ratings of perceived exertion	70%Δ	13.8 [12.9–14.7]	1.6 [1.3–1.9]	– 0.08 [– 0.12 to – 0.06]	N/A	1.6 [1.0–2.2]	1.0 [0.8–1.2]	73.5 [49.4–84.9]	26.5 [14.8–48.2]
	85%$\dot{\text{W}}$_max_	13.1 [11.5–14.6]	3.5 [2.2–4.9]	– 0.55 [– 0.94 to – 0.25]	0.03 [0.01–0.06]	1.4 [0.8–2.1]	1.2 [0.9–1.5]	57.3 [26.3–78.9]	42.7 [21.0–72.0]
	120%TT	14.0 [12.7–15.2]	2.1 [1.6–2.5]	– 0.12 [– 0.16 to – 0.07]	N/A	2.3 [1.4–3.3]	1.0 [0.8–1.2]	83.8 [65.1–92.9]	16.2 [7.0–32.2]
	80%W'	13.1 [11.2–14.6]	3.5 [2.3 – 4.8]	– 0.55 [– 0.90 to – 0.26]	0.03 [0.01–0.05]	1.4 [0.8–2.1]	1.2 [0.9–1.5]	57.3 [29.2–78.6]	42.7 [21.0–69.8]
Blood lactate concentration (mmol·L^−1^)	70%Δ	4.4 [2.2–6.4]	5.0 [3.6–6.3]	– 0.9 [– 1.2 to – 0.6]	0.04 [0.03–0.06]	2.9 [1.9–3.9]	1.6 [1.3–1.8]	77.8 [57.2–87.6]	22.2 [12.2–42.8]
	85%$\dot{\text{W}}$_max_	5.5 [3.6–7.1]	4.9 [3.8–6.1]	– 0.9 [– 1.1 to – 0.6]	0.05 [0.03–0.06]	3.1 [2.0–4.1]	1.1 [1.0–1.3]	87.8 [74.2–93.6]	12.2 [5.9–25.4]
	120%TT	5.5 [3.1–7.8]	5.9 [4.0–8.0]	– 1.1 [– 1.7 to – 0.7]	0.06 [0.03–0.10]	3.3 [2.1–4.4]	1.6 [1.3–1.9]	79.7 [60.5–90.3]	20.3 [9.5–38.4]
	80%W'	9.3 [7.2–11.1]	2.8 [2.0 – 3.4]	– 0.3 [– 0.4 to – 0.2]	N/A	3.2 [1.9–4.2]	1.7 [1.3–2.0]	78.5 [51.0–89.5]	21.5 [10.5–47.6]
Heart rate (beats·min^−1^)	70%Δ	161 [154–168]	5 [3–7]	– 0.4 [– 0.6 to – 0.2]	N/A	12 [7–16]	6 [5–7]	77.0 [55.9–87.2]	23.0 [12.6–43.2]
	85%$\dot{\text{W}}$_max_	157 [147–165]	10 [4–14]	– 1.9 [– 3.1 to – 0.6]	0.1 [0.0–0.2]	12 [8–17]	6 [5–7]	80.2 [61.4–90.0]	19.8 [9.7–37.5]
	120%TT	161 [153–167]	5 [2–6]	– 0.3 [– 0.5 to – 0.1]	N/A	15 [9–21]	4 [3–5]	93.7 [85.6–97.1]	6.3 [2.8–14.3]
	80%W'	164 [158–170]	4 [2–5]	– 0.3 [– 0.5 to 0.0]	N/A	11 [8–15]	5 [3–5]	85.3 [72.0–93.2]	14.7 [6.4–26.7]
Ventilation (L·min^−1^)	70%Δ	125 [115–136]	9 [4–12]	– 0.6 [– 0.9 to – 0.1]	N/A	19 [13–26]	12 [9–13]	72.8 [50.8–83.5]	27.2 [16.1–47.3]
	85%$\dot{\text{W}}$_max_	133 [120–146]	7 [2–12]	– 0.5 [– 0.9–0.0]	N/A	20 [12–27]	11 [9–13]	74.7 [49.2–87.2]	25.3 [12.8–48.8]
	120%TT	131 [118–143]	9 [3–15]	– 0.7 [– 1.4 to – 0.2]	N/A	20 [14–29]	13 [10–15]	71.3 [49.0–86.8]	28.7 [12.9–49.5]
	80%W'	138 [128–151]	7 [3–10]	– 0.5 [– 0.8 to 0.0]	N/A	22 [14–29]	9 [7–11]	84.2 [67.2–92.8]	15.8 [7.1–31.7]
Respiratory frequency (cycles·min^−1^)	70%Δ	43 [38–48]	5 [3–6]	– 0.2 [– 0.3 to – 0.1]	N/A	12 [8–15]	4 [4–5]	87.9 [74.9–92.6]	12.1 [7.4–24.3]
	85%$\dot{\text{W}}$_max_	45 [39–51]	5 [4–7]	– 0.4 [– 0.5 to – 0.2]	N/A	10 [7–13]	4 [3–4]	88.4 [72.6–93.3]	11.6 [6.5–25.5]
	120%TT	39 [33–46]	13 [8–17]	– 2.2 [– 3.4 to – 1.1]	0.1 [0.0–0.2]	11 [7–14]	4 [3–4]	89.3 [78.0–95.0]	10.7 [5.0–21.0]
	80%W'	43 [36–49]	9 [5–14]	– 1.4 [– 2.6 to – 0.5]	0.1 [0.0–0.1]	11 [7–15]	4 [3–4]	90.8 [80.2–96.1]	9.2 [3.8–18.7]
ΔStO_2_ (%)	70%Δ	– 21.4 [– 24.5 to – 18.3]	– 0.4 [– 0.6 to – 0.1]	N/A	N/A	7.0 [4.3–9.5]	2.8 [2.3–3.2]	86.5 [68.9–93.2]	13.5 [6.7–30.1]
	85%$\dot{\text{W}}$˙_max_	– 20.9 [– 23.3 to – 17.9]	– 0.3 [– 0.5 to – 0.1]	N/A	N/A	5.1 [3.2–6.7]	1.4 [1.1–1.6]	93.2 [84.1–96.5]	6.8 [3.4–15.8]
	120%TT	– 21.7 [– 25.5 to – 17.4]	1.5 [– 0.3–3.1]	– 0.4 [– 0.7 to – 0.1]	N/A	6.8 [4.1–9.9]	1.7 [1.3–2.1]	94.3 [85.1–97.6]	5.7 [2.2–14.9]
	80%W'	– 19.4 [– 22.3 to – 16.6]	– 0.5 [– 0.7 to – 0.3]	N/A	N/A	5.7 [3.7–7.6]	1.3 [1.0–1.6]	95.3 [88.1–97.9]	4.7 [2.1–11.5]
Δdeoxy[heme] (µm)	70%Δ	84 [69–100]	1.5 [0.6–2.3]	N/A	N/A	32 [20–42]	10 [8–11]	91.4 [80.6–95.2]	8.6 [4.7–18.6]
	85%$\dot{\text{W}}$_max_	80 [69–95]	1.1 [0.2–1.8]	N/A	N/A	21 [13–28]	6 [5–7]	92.7 [80.1–96.3]	7.3 [3.5–18.8]
	120%TT	79 [64–91]	1.7 [0.3–3.0]	N/A	N/A	29 [19–39]	5 [4–6]	97.3 [93.0–98.9]	2.7 [1.1–7.0]
	80%W'	82 [67–96]	1.9 [0.7–3.0]	N/A	N/A	32 [21–44]	6 [5–7]	96.3 [91.4–98.2]	3.7 [1.6–8.0]
Cadence (rev·min^−1^)	70%Δ	102 [98–107]	– 2 [– 2 to – 1]	N/A	N/A	9 [6–12]	5 [4–6]	74.7 [53.6–85.3]	25.3 [14.7–45.6]
	85%$\dot{\text{W}}$_max_	101 [97–105]	– 2 [– 2 to – 1]	N/A	N/A	8 [5–11]	6 [4–6]	74.3 [48.6–87.3]	25.7 [12.3–51.0]
	120%TT	100 [94–104]	– 2 [– 3 to – 1]	N/A	N/A	9 [5–13]	6 [5–7]	68.9 [43.0–83.3]	31.1 [16.6–56.3]
	80%W'	100 [95–105]	– 2 [– 3 to – 1]	N/A	N/A	9 [6–12]	5 [4–6]	77.0 [55.9–89.0]	23.0 [10.9–43.8]

Formula: dependent variable = intercept + interval coefficient_(a)_ · *x* + interval coefficient_(b)_ · *x*^2^ + interval coefficient_(c)_ · *x*^3^, where *x* = work interval number. ΔStO_2_, changes from a resting baseline in muscle tissue oxygen saturation; Δdeoxy[heme], changes from a resting baseline in the concentration of deoxygenated heme compounds; N/A, not applicable (i.e., consider the coefficient as 0). See text for intensity prescription abbreviations

**Table 3:** Central tendency and dispersion measures for time > 90%$\dot{\text{V}}$O_2max_ and time > 95%$\dot{\text{V}}$O_2max_ [95% confidence limits]

Intensity prescription	Time > 90%$\dot{\text{V}}$O_2max_ (s)		Time > 95%$\dot{\text{V}}$O_2max_ (s)		Time > 90%$\dot{\text{V}}$O_2max[%TTE]_		Time > 95%$\dot{\text{V}}$O_2max[%TTE]_	
	Median	Q1–Q3	Median	Q1–Q3	Mean	SD	Mean	SD
70%Δ	478*	346–633	143	65–345	32.8 [25.1–40.5]	15.5 [11.7–23.3]	17.4 [9.7–25.0]	15.4 [11.5–23.1]
85%$\dot{\text{W}}$_max_	385	300–720	168	83–439	36.5 [29.6–43.3]	13.8 [10.3–20.7]	19.0 [11.9–26.0]	14.2 [10.7–21.3]
120%TT	275	113–430	148	15–225	32.2 [22.7–41.8]	19.3 [14.5–28.9]	16.5 [9.5–23.5]	14.1 [10.6–21.1]
80%W'	338	141–434	98	44–255	36.1 [28.1–44.1]	16.0 [12.0–24.0]	19.7 [11.7–27.7]	16.0 [12.0–24.0]

Q1, 25th percentile; Q3, 75th percentile. * denotes difference from 120%TT (*P* = 0.014). See text for intensity prescription abbreviations

## Discussion

This study focused on the methodological aspect of intensity prescription for HIIT. By assessing inter-individual variability in performance, physiological and perceptual responses to HIIT sessions randomly prescribed to cyclists at 70%Δ, 85%$\dot{\text{W}}$_max_, 120%TT, and 80%W', it sought to identify the optimal approach to normalise exercise intensity. In other words, it was expected that at least one prescription method would minimise the magnitude of inter-individual variability in acute HIIT responses. However, it was not possible to detect clear evidence, be it performance-related, physiological, or perceptual, to support the use of one method over the others. When log-transformed time to exhaustion was modelled as a function of exercise intensity, a similarly high magnitude of inter-individual variability was observed for all normalisation methods. Given the pooled median time to exhaustion of 15.3 min, and wide interquartile ranges of 24.1, 19.0, 12.6, and 12.6 min for, respectively, 70%Δ, 85%$\dot{\text{W}}$_max_, 120%TT, and 80%W', these intensity normalisation methods may be considered ineffective for prescription of HIIT.

### Methodological aspects

Previous studies investigating exercise intensity normalisation (McLellan and Skinner 1985; Meyer et al. 1999; Baldwin et al. 2000; Scharhag-Rosenberger et al. 2010; Lansley et al. 2011; Egger et al. 2016; Coyle et al. 1988; Iannetta et al. 2020; Julio et al. 2020; Galbraith et al. 2015; Ferguson et al. 2013; Bartram et al. 2018) can be categorised according with their experimental design, from the least to most robust approach for the evaluation of a method: (a) individualised work rate targets based on percentages of a maximal benchmark (e.g., 70% $\dot{\text{V}}$O_2max_, 60%$\dot{\text{W}}$_max_) are expressed relative to a criterion intensity-domain transition marker (e.g., %$\dot{\text{W}}$_GET_, %CP), with resultant variability quantified (Meyer et al. 1999; Iannetta et al. 2020); (b) bouts of exercise are performed at work rates normalised to one or more benchmarks, with raw variability in individual exercise responses, or agreement between predicted and actual responses, quantified (Baldwin et al. 2000; Scharhag-Rosenberger et al. 2010; Lansley et al. 2011; Coyle et al. 1988; Bartram et al. 2018; Ferguson et al. 2013; Galbraith et al. 2015; Julio et al. 2020); (c) exercise responses at multiple timepoints or conditions are modelled as a function of different benchmarks to minimise the influence of random variability over estimates of inter-individual variability (present study, McLellan and Skinner (1985), and Egger et al. (2016)). While methodological differences preclude direct comparison of our results with those of other studies, it was possible to draw general conclusions (see subsequent sections) by reanalysing raw data directly available in tables or through data extraction from figures with WebPlotDigitizer (http://automeris.io/WebPlotDigitizer). Inter-individual variability was quantified as SD or CV. If time to exhaustion with mean *t* is considered, it is expected that approximately 68.2% of the individuals sampled from a population will reach exhaustion in between *t* – SD and *t* + SD, or between *t* – CV_(%*t*)_ and *t* + CV_(%*t*)_. For example, if *t* = 1000 s, and SD = 400 s, CV will be 40%. Hence, approximately 68.2% of the individuals sampled from a population will reach exhaustion in between 600 and 1400 s. Being the CV a percentage, it is sometimes possible to extrapolate a given estimate to other samples with different means.

### Performance variability

Several authors have recommended that %$\dot{\text{V}}$O_2max_, the traditional approach to normalising exercise intensity, is abandoned (McLellan and Skinner 1985; Meyer et al. 1999; Baldwin et al. 2000; Scharhag-Rosenberger et al. 2010; Lansley et al. 2011; Egger et al. 2016; Coyle et al. 1988; Iannetta et al. 2020; Jamnick et al. 2020; Mann et al. 2013; Rossiter 2011). In this study, with HIIT performed at approximately 92.3% $\dot{\text{V}}$O_2max_, the inter-individual CV for log-transformed time to exhaustion was 50.4%. This figure suggests that HIIT normalised to % $\dot{\text{V}}$O_2max_ may elicit similar or slightly greater performance variability than constant-intensity exercise, given the inter-individual CVs of 42.8, 43.4, 42.5, and 41.8% estimated for constant-intensity exercise at approximately 75% (Scharhag-Rosenberger et al. 2010), 88.2% (Coyle et al. 1988), 90% (Lansley et al. 2011), and 94.8%$\dot{\text{V}}$O_2max_ (McLellan and Skinner 1985), respectively. While the present study reinforces the consensual view about %$\dot{\text{V}}$O_2max_, none of the alternative methods evaluated performed better (see Table 1). In contrast to what has been shown for constant-intensity exercise, in which the %Δ method may decrease performance variability (McLellan and Skinner 1985; Lansley et al. 2011), our inter-individual CVs for log-transformed time to exhaustion varied from 44.2% (%$\dot{\text{W}}$_max_) to 59.1% (%TT), with confidence intervals of all prescriptions overlapping substantially, and lower limits of approximately 30%.

A popular approach to normalising exercise intensity for HIIT consists of using individuals’ CP and W' (Ferguson et al. 2013; Galbraith et al. 2015; Bartram et al. 2018; Jones and Vanhatalo 2017). Ferguson et al. (2013) asked eight active men to perform three HIIT sessions (running) to exhaustion, all with four-minute work and recovery intervals (HIIT_4min/4 min_), at work rates predicted to expend 100%W' in 4, 6, and 8 min. The inter-individual CVs for time to exhaustion were 20.3, 20.3, and 22.8%, respectively. Even with a similar HIIT format and total exercise duration, our study does not corroborate Ferguson’s findings (Ferguson et al. 2013), given the CV of 49.2% for log-transformed time to exhaustion as a function of %W'. A likely reason for the discrepancy resides in the fact that a 3-min all-out test was used in the present study to avoid excessive participant burden, whereas Ferguson et al. (2013) adopted four constant-work rate bouts to determine CP and W'. While the 3 min all-out test was initially considered valid (Vanhatalo et al. 2007), more recent studies have questioned its use with trained cyclists due to inaccurate predictions (Nicolò et al. 2017; Bartram et al. 2017). Interestingly, Julio et al. (2020) have shown that the inter-individual CV for running time to exhaustion can be reduced from 45.2 to 21.8% when HIIT with fifteen-second work and recovery intervals (HIIT_15s/15 s_) is prescribed relative to the anaerobic speed reserve rather than $\dot{\text{W}}$_max_. Taken together, the results of Ferguson et al. (2013) and Julio et al. (2020) suggest that an inter-individual CV of approximately 20% for time to exhaustion is an achievable target for HIIT, although several popular methods of exercise intensity normalisation (i.e., % $\dot{\text{V}}$O_2max_, %Δ, %$\dot{\text{W}}$_max_, %TT, and %W' based on a 3-min all-out test) may fail to produce such an outcome.

### Physiological variability

From a physiological standpoint, [La^−^] responses to HIIT varied substantially between individuals, with no between-condition differences in magnitude to suggest there was an optimal method for exercise intensity normalisation. Here, a CV would be less intelligible due to the rising pattern of exercise responses after each work interval (see interval coefficients in Table 2). Nevertheless, previous studies have frequently used [La^−^] as a marker of metabolic stress (Scharhag-Rosenberger et al. 2010; Coyle et al. 1988; Lansley et al. 2011; Meyer et al. 1999; Baldwin et al. 2000; Egger et al. 2016; Julio et al. 2020; Ferguson et al. 2013), providing a reference for our numbers. The inter-individual SD for [La^−^] during HIIT varied from 2.9 (70%Δ) to 3.3 mmol·L^−1^ (120%TT), with lower confidence limits of approximately 2.0 mmol·L^−1^ for all prescriptions. Coyle et al. (1988) reported [La^−^] of 11.0 ± 4.4 mmol·L^−1^ immediately after exhaustion when cyclists performed constant-intensity exercise at 88.2%$\dot{\text{V}}$O_2max_. Julio et al. (2020) reported [La^−^] changes from resting to exhaustion of 7.7 ± 3.4 mmol·L^−1^ when HIIT_15s/15 s_ was prescribed as 110%$\dot{\text{W}}$_max_. While these figures indicate the levels of inter-individual variability found in our study are not unusual for high-intensity exercise, the data obtained by Ferguson et al. (2013) are remarkably more homogenous. Their three HIIT_4min/4 min_ sessions, at work rates predicted to expend 100%W' in 4, 6, and 8 min, led to [La^−^] at exhaustion of 9.7 ± 1.1, 8.5 ± 1.5, and 8.0 ± 1.1 mmol·L^−1^, respectively. Indeed, Ferguson et al. (2013) SDs are just slightly higher than those modelled by Egger et al. (2016) across different submaximal intensities and taking into account intra-individual variability (i.e., 0.6, 0.9, 0.4, and 0.5 mmol·L^−1^ for exercise intensity expressed as %$\dot{\text{V}}$O_2max_, %oxygen uptake reserve, %maximal heart rate, and %heart rate reserve, respectively); and comparable to those reported by studies in which constant-intensity exercise was performed at a much lower intensity (i.e., 75% $\dot{\text{V}}$O_2max_), with [La^−^] of 4.6 ± 1.9 (Scharhag-Rosenberger et al. 2010) and 2.8 ± 1.1 mmol·L^−1^ (Meyer et al. 1999). It may be speculated that using CP and W' determined from four bouts of constant-work rate exercise, as employed by Ferguson et al. (2013), minimises inter-individual variability in [La^−^] in addition to time to exhaustion. However, this possibility should be scrutinised in light of Ferguson et al. (2013) sample of only eight individuals.

Given that [La^−^] plays a central role in the coordination of metabolic responses to exercise (Ferguson et al. 2018), it is unsurprising that most studies on exercise intensity normalisation have drawn conclusions from the inter-individual variability in [La^−^] as a marker of exercise intensity (Scharhag-Rosenberger et al. 2010; Lansley et al. 2011; Meyer et al. 1999; Baldwin et al. 2000; Egger et al. 2016; Julio et al. 2020). However, in the present study, only 27% of time to exhaustion variability was accounted for by [La^−^] measured at 4 min (i.e., at the end of the first work interval), whereas, for constant-intensity bouts, 59% (McLellan and Skinner 1985) and 75% (Sassi et al. 2006) of time to exhaustion variability was accounted for by [La^−^] measured at 6 and 10 min, respectively. Our estimate, therefore, reinforces the need for a multivariate approach to investigate exercise intensity normalisation (Egger et al. 2016), particularly in the context of HIIT.

Cardiorespiratory responses tend not to achieve steady-state during HIIT sessions as employed in this study, increasing continuously towards maximal values within and between successive work intervals (Rossiter 2011). For this reason, a snapshot of responses elicited by each four-minute work interval was obtained by averaging measures recorded from the third to the fourth minute, although some information is lost (i.e., for modelling purposes) with this approach. Heart rate, ventilation, and respiratory frequency increased after each work interval (see interval coefficients in Table 2), approaching maximal values near exhaustion, consistent with exercise responses to constant-intensity bouts performed in the very heavy domain (Horstman et al. 1979; Marcora and Staiano 2010; Lansley et al. 2011; Rossiter 2011). However, all model parameters were very similar between HIIT sessions, providing no evidence for an optimal intensity normalisation method.

Interestingly, work interval number did not affect oxygen uptake, suggesting that most participants reached a high % $\dot{\text{V}}$O_2max_ from the first work interval onwards. Indeed, intercepts for oxygen uptake varied from 50.1 (120%TT) to 51.2 ml·kg^−1^·min^−1^ (80%W'), representing 92.3% and 94.3% $\dot{\text{V}}$O_2max_, respectively. Inter-individual variability was also similar across HIIT sessions, with SDs varying from 6.6 (70%Δ) to 8.3 ml·kg^−1^·min^−1^ (120%TT), and overlapping confidence intervals. From a training perspective, exercise time at or near $\dot{\text{V}}$O_2max_ has been used as a marker of the adaptive potential of HIIT sessions, based on the premise that such intensities impose maximum stress on the physiological processes and structures determining $\dot{\text{V}}$O_2max_ (Buchheit and Laursen 2013). In this regard, 70%Δ elicited a longer time > 90% $\dot{\text{V}}$O_2max_ compared with 120%TT in the current study (see Table 3), likely due to a longer time to exhaustion compared with both 120%TT and 80%W' (see Fig. 3). The difference nevertheless disappeared and data became remarkably similar across HIIT sessions when time > 90% $\dot{\text{V}}$O_2max_ was expressed relative to time to exhaustion, both in terms of means and SDs. Altogether, the evidence refutes the hypothesis that oxygen uptake responses to HIIT can be better normalised with one prescription method versus another, as demonstrated for constant-intensity exercise (Lansley et al. 2011).

The adoption of near-infrared spectroscopy to assess inter-individual variability in tissue oxygenation of the vastus lateralis muscle is unique to the present study. Specifically, Δdeoxy[heme] represents the extent to which oxygen is extracted from the perfusing blood, whereas ΔStO_2_ represents the relative balance between oxygen delivery and uptake (Barstow 2019). Accordingly, Δdeoxy[heme] increases and ΔStO_2_ decreases as exercise intensity increases, although these relationships are not linear (Boone et al. 2016; Stöcker et al. 2017). In contrast to oxygen uptake, Δdeoxy[heme] slightly increased and ΔStO_2_ slightly decreased after each work interval, suggesting a progressive deterioration of oxygen delivery as per the Fick principle (Fick 1870), presumably due to cardiac output redistribution towards respiratory muscles (Harms et al. 1997; Turner et al. 2013). The increments in ventilation and respiratory frequency observed after each work interval support this interpretation. Regardless, all tested methods of exercise intensity normalisation elicited similar inter-individual SDs, with overlapping confidence intervals. Therefore, it remains to be determined whether near-infrared spectroscopy, with all of its methodological challenges (Barstow 2019), would be sensitive to quantifying inter-individual variability in muscle tissue oxygenation.

### Perceptual variability

While it is most common to investigate performance variability from a physiological point of view (Bossi et al. 2017; McLellan and Skinner 1985; Sassi et al. 2006), RPE has been shown to predict time to exhaustion during constant-intensity bouts performed in the very heavy domain (Horstman et al. 1979; Marcora and Staiano 2010). For instance, Marcora and Staiano (2010) identified that 67% of time to exhaustion variability was accounted for by RPE measured at 8 min. In contrast, RPE at 4 min (i.e., at the end of the first work interval) accounted for only 12% of time to exhaustion variability in the present study, suggesting that performance during HIIT may be more unpredictable compared with constant-intensity bouts.

Lansley et al. (2011) have previously shown that inter-individual variability in RPE at 5 min can be reduced when constant-intensity exercise is performed at 80%Δ (18 ± 1) as opposed to 90%$\dot{\text{V}}$O_2max_ (19 ± 2). However, these measures were obtained too close to exhaustion (i.e., 8.6 ± 1.8 and 5.4 ± 2.3 min, respectively), constraining inter-individual variability as ratings were bounded to 20. This makes it difficult to compare our results with those of Lansley et al. (2011). It is interesting, however, that across all variables of interest, modelled RPE produced the lowest inter-individual SDs relative to the standard error of estimate (see Table 2). While it is conceivable that RPE as a marker of exercise intensity could be more sensitive than other physiological variables, none of the normalisation methods investigated appeared to be preferable, with SDs varying from 1.4 (85%$\dot{\text{W}}$_max_ and 80%W') to 2.3 (120%TT) and overlapping confidence intervals. Thus, further research is required to validate the use of RPE as a tool to investigate the best methods of exercise intensity normalisation.

### Implications

As exercise intensity purportedly regulates both acute and chronic (i.e., adaptive) responses to, respectively, single and repeated bouts of exercise (MacInnis and Gibala 2017; Granata et al. 2018; Wenger and Bell 1986), physiologists have tried to identify optimal approaches for its normalisation (Mann et al. 2013; Jamnick et al. 2020). While the present study does not offer a solution in this regard, there are several implications. For instance, the large inter-individual variability in the relationship between intensity and time to exhaustion during HIIT, irrespective of how intensity was expressed, poses a challenge to practitioners and researchers. Consider this hypothetical scenario: a coach prescribes HIIT sessions for $\dot{\text{V}}$O_2max_ enhancement to three cyclists, with six 4 min work intervals at 80%$\dot{\text{W}}$_max_. Cyclist one repeatedly fails to complete the sessions at the target work rate, cyclist two finds the sessions too easy, and cyclist three completes the sessions at the very limit of tolerance. Besides the fact that cyclists one and two might question the coach’s ability to appropriately prescribe HIIT, it is possible that only cyclist three will manifest the desired adaptive effect (i.e., $\dot{\text{V}}$O_2max_ increase), based on the premise that the magnitude of adaptive responses reflects, at least partially, the magnitude of the training stimulus (Mann et al. 2014; Flück 2006; Perry et al. 2010). Another possible problem may arise when scientists prescribe HIIT to a group of research participants and some are unable to complete the session, potentially leading to their exclusion from the sample, and ultimately biasing estimates of variables under investigation. Finally, our findings are in line with the contention that some of the inter-individual variability in adaptive responses following HIIT programmes (Astorino and Schubert 2014; Coakley and Passfield 2018; Montero and Lundby 2017; Williams et al. 2019) may result from how intensity was normalised across participants (Mann et al. 2014, 2013; Iannetta et al. 2020; Jamnick et al. 2020).

From an analytical perspective, any gross estimate of inter-individual variability (i.e., not modelled) is subject to overestimation because it does not take into account measurement error and day-to-day biological variability (Egger et al. 2016). To address this requirement, multiple timepoints or conditions should be modelled together, and at least one condition should be repeated (Egger et al. 2016). While in the current study, the repetition requirement was achieved for time to exhaustion by expressing the four target work rates of each participant as %$\dot{\text{V}}$O_2max_, %Δ, %$\dot{\text{W}}$_max_, %TT, and %W', this was not the case for physiological and perceptual responses. This means that modelled SDs for these latter responses did not account for day-to-day variability, potentially overestimating the true inter-individual variability elicited by each method of intensity prescription. However, modelled inter-individual estimates are rare in the literature, with few studies using this approach (Egger et al. 2016; McLellan and Skinner 1985). Thus, the higher unpredictability of acute HIIT responses in this investigation compared with referenced studies is not merely the result of statistical artefacts.

### Limitations

Despite the best efforts to eliminate potential sources of methodological bias, the results of this study’s pilot work were slightly skewed in the sense that 70%Δ ended up being a lower exercise intensity (in watts) compared with 85%$\dot{\text{W}}$_max_, 120%TT, and 80%W'. Unsurprisingly, time to exhaustion was longer compared with 120%TT and 80%W'. However, there is no evidence to suggest that results would have been different had HIIT been prescribed at 75%Δ rather than 70%Δ. Besides, from a subjective perspective, performance was rated poorer for 120%TT compared with 70%Δ and 85%$\dot{\text{W}}$_max_, and frustration was rated higher for 80%W' compared with 70%Δ and 85%$\dot{\text{W}}$_max_, likely as a consequence of the short median times to exhaustion of these conditions. As participants were blinded to the target power outputs, some were clearly disappointed at the end of their “training sessions”. Nevertheless, it would have been very difficult to demand that all participants exercised to exhaustion had the relative intensity of all prescriptions been decreased. Indeed, in five HIIT sessions (out of seventy-two), participants completed ten work intervals (the pre-set maximum), although in all these instances they rated an RPE of 20 for the last work interval, suggesting they were very close to exhaustion. In terms of sample size, a much larger number of participants would increase the accuracy of the linear mixed model estimates. Although potential between-method differences in the magnitude of inter-individual variability could become evident, given the observed data, it is very unlikely that one of the methods investigated would then arise as valid for HIIT prescription.

## Conclusions

The evidence reported in this study suggests that methods of intensity prescription that are often deemed scientifically valid do not normalise acute responses to HIIT between individuals. Time to exhaustion as a measure of HIIT performance, [La^−^], RPE, cardiorespiratory responses, and muscle tissue oxygenation were all equally variable between individuals when expressed as %Δ, %$\dot{\text{W}}$_max_, %TT, or %W'. Further studies are required to determine the optimal approach for exercise intensity normalisation of HIIT.

## Data Availability

The datasets generated during and/or analysed during the current study are available from the corresponding author on reasonable request.

## Abbreviations

%TT: Fraction of the mean work rate of a twenty-minute time-trial
%$\dot{\text{V}}$O_2max_: Fraction of an individual’s maximal oxygen uptake
%Δ: Fraction of the difference between the work rate associated with gas exchange threshold and the work rate associated with maximal oxygen uptake
[La^−^]: Blood lactate concentration
CP: Critical power
CV: Coefficient of variation
F: Ratio of explained variance to unexplained variance
F_r_: Statistical score of Friedman analysis of variance
GET: Gas exchange threshold
HIIT: High-intensity interval training
HIIT_15s/15__ s_: High-intensity interval training with fifteen-second work and recovery intervals
HIIT_4min/4__ min_: High-intensity interval training with four-minute work and recovery intervals
NASA-TLX: National Aeronautics and Space Administration Task Load Index
ƞ_p_^2^: Partial eta-squared effect size
P: Probability of obtaining the observed results assuming that the null hypothesis is true
RPE: Ratings of perceived exertion
SD: Standard deviation
sRPE: Session ratings of perceived exertion
Time > 90%$\dot{\text{V}}$O_2max_: Accumulated exercise time with oxygen uptake above ninety percent of maximal oxygen uptake
Time > 90%$\dot{\text{V}}$O_2max__[%TTE]_: Accumulated exercise time with oxygen uptake above ninety percent of maximal oxygen uptake as a percentage of time to exhaustion
Time > 95%$\dot{\text{V}}$O_2max_: Accumulated exercise time with oxygen uptake above ninety-five percent of maximal oxygen uptake
Time > 95%$\dot{\text{V}}$O_2max__[%TTE]_: Accumulated exercise time with oxygen uptake above ninety-five percent of maximal oxygen uptake as a percentage of time to exhaustion
Var: Variance
$\dot{\text{V}}$O_2max_: Maximal oxygen uptake
W': Work capacity above critical power
$\dot{\text{W}}$_GET_: Work rate associated with gas exchange threshold
$\dot{\text{W}}$_max_: Maximal work rate of an incremental test to exhaustion
$\dot{\text{W}}$_target_: Work rate prescribed for high-intensity interval training
Δdeoxy[heme]: Changes in concentration of deoxygenated heme compounds
ΔStO_2_: Changes in muscle tissue oxygen saturation
